# The right to be heard: exploring epistemic injustice in dementia care—A qualitative study

**DOI:** 10.1093/geront/gnag219

**Published:** 2026-09-01

**Authors:** Lara Calabrese, Marco Brigiano, Martina Quartarone, Alice Annini, Elisa Ferriani, Stefania Martinelli, Lisa Bortolotti, Rabih Chattat

**Affiliations:** Department of Psychology, University of Bologna, Bologna, Italy; Department of Psychology, University of Bologna, Bologna, Italy; Department of Psychology, University of Bologna, Bologna, Italy; Department of Psychology, University of Bologna, Bologna, Italy; Hospital Psychology Unit, Azienda Unità Sanitaria Locale (AUSL) of Bologna, Bologna, Italy; L’Aquilone Social Cooperative, Savignano sul Rubicone, Forlì-Cesena, Italy; Department of Philosophy, University of Birmingham, Birmingham, United Kingdom; Department of Neurosciences and Rehabilitation, University of Ferrara, Ferrara, Italy; Department of Psychology, University of Bologna, Bologna, Italy

**Keywords:** Dementia, Epistemic injustice, Epistemic agency, Thematic analysis, Qualitative research

## Abstract

**Background and Objectives:**

Epistemic injustice, introduced by philosopher Miranda Fricker in 2007, refers to harm affecting individuals as knowers because of social prejudices and stereotyping. This leads to the partial or complete exclusion of certain individuals from knowledge building. People living with dementia are particularly vulnerable because cognitive impairments may reinforce assumptions about reduced credibility, marginalizing their voices in clinical, social, and family contexts and limiting opportunities to exercise epistemic agency. This research examines how people living with dementia experience epistemic injustice in everyday life and how it shapes their participation as knowers in clinical, social, and family contexts.

**Research Design and Methods:**

A qualitative study using semi-structured interviews with a conversational approach was conducted. Participants (*N* = 15) with a dementia diagnosis were interviewed in their homes or nursing homes. Interviews were audio-recorded, transcribed, and analyzed thematically to identify recurring patterns and meanings.

**Results:**

Three interconnected domains were identified: epistemic marginalization in clinical encounters, everyday negotiation of competence and agency, and epistemic invalidation and silencing in interpersonal relationships. Participants often perceived these experiences as appropriate or expected rather than unjust, reflecting internalized stigma. Many felt their perspectives were no longer recognized as valuable, leading to withdrawal from care and communication and fewer opportunities to contribute their knowledge.

**Discussion and Implications:**

Epistemic injustice extends beyond healthcare, affecting everyday and family interactions by progressively limiting opportunities for people living with dementia to participate as credible knowers. Its normalization fosters self-silencing, perpetuating disempowerment, and social exclusion.

Dementia is a major neurocognitive disorder characterized by progressive impairments in cognitive functions such as memory, language, and information processing, which can significantly affect individuals’ ability to communicate and participate in everyday interactions (Spencer, 2023). These disease-related changes place people living with dementia among the social groups most at risk of marginalization and exclusion from knowledge production and decision-making processes (Young et al., 2019). This vulnerability is particularly concerning given the rapidly growing global prevalence of dementia, which is expected to increase substantially over the coming decades (Alzheimer’s Disease International, 2024).

Originally introduced by philosopher Miranda Fricker (2007), epistemic injustice refers to a form of harm inflicted on individuals in their capacity as knowers, whereby they are denied credibility or lack access to shared interpretative resources. According to Fricker (2007), epistemic justice is closely connected to the right to be heard and to have one’s testimony assessed fairly, without credibility being diminished because of prejudice. While no individual has a right to be believed unconditionally, listeners have a responsibility to evaluate a speaker’s credibility based on available evidence regarding their sincerity and reliability, rather than on prejudicial assumptions. When credibility is unjustly reduced because of prejudice—such as negative overgeneralizations about cognitive capacities associated with a diagnosis of dementia—testimonial injustice occurs. In the context of dementia, epistemic injustice commonly manifests through testimonial injustice, which arises when a speaker’s credibility is unjustly deflated due to the listener’s prejudicial biases (Spencer, 2023), and hermeneutical injustice, which occurs when individuals or groups struggle to communicate their social experiences because society lacks the interpretative frameworks to understand them (Halonen et al., 2025). In everyday life, these forms of injustice may manifest when people living with dementia are spoken for rather than spoken to, when their accounts and preferences are afforded less credibility because of assumptions associated with the diagnosis, or when they are excluded from conversations and decision-making on the presumption that they have little meaningful contribution to offer. Over time, such experiences may progressively undermine opportunities to exercise epistemic agency and participate as credible knowers in clinical, family, and social contexts.

Although these processes may coexist with broader experiences of stigma, discrimination, or social exclusion, they should not be considered synonymous. Social exclusion concerns reduced participation in social life more generally, whereas epistemic injustice specifically concerns the denial of a person’s status as a credible knower and contributor to shared understanding (Fricker, 2007).

Social stigma plays a central role in shaping how dementia is understood and responded to, often reinforcing stereotypes that portray people living with dementia as entirely dependent, cognitively incompetent and unable to contribute meaningfully to conversations about their own lives (Hermann et al., 2018). In the context of dementia, dementia-related stigma refers to the negative stereotypes, prejudices, and discriminatory behaviors directed toward people living with dementia, their caregivers and their families (Brookman et al., 2025; Hermann et al., 2018; Low & Purwaningrum, 2020). Such representations not only influence public attitudes but may also be internalized by individuals with dementia, leading to reduced self-esteem, social withdrawal and diminished participation (Young et al., 2019). In this context, epistemic injustice provides a useful theoretical framework for understanding how stigma and prejudice become translated into concrete interpersonal processes through which people living with dementia may be denied credibility, excluded from knowledge exchanges, or discouraged from participating in decisions affecting their own lives.

Research has increasingly framed these issues within a broader rights-based perspective. Moving beyond traditional person-centered approaches, Bartlett and O’Connor (2007) proposed a citizenship framework that recognizes people living with dementia as active citizens with rights, responsibilities, and opportunities to participate in society, including the production of knowledge about their own lives. Subsequent developments of the citizenship approach have further emphasized that people living with dementia should be recognized not only as recipients of care but also as active contributors to research, policy, and practice (Bartlett & O’Connor, 2010). From this perspective, meaningful involvement in research is not merely a methodological preference but a matter of social justice, democratic participation and recognition of people living with dementia as legitimate epistemic contributors (Bartlett & O’Connor, 2010).

This perspective is further reinforced by the Alzheimer’s Europe position paper (Gove et al., 2018), which explicitly affirms the right of people living with dementia to participate in research and in decisions concerning research priorities, recognizing their experiential knowledge as indispensable for developing policies, services and scientific evidence that reflect their needs and preferences. Given that, to best of our knowledge, no validated interview protocols or assessment tools currently exist to explore experiences of epistemic injustice among people living with dementia, these conceptual and rights-based frameworks informed the development of the interview guide used in this study by identifying everyday domains relevant to epistemic participation, credibility, communication and involvement in decision-making, while allowing participants to describe these experiences in their own words.

Although interest in epistemic injustice within health and psychological research has grown in recent years, studies in the dementia field have largely relied on the perspectives of clinicians, caregivers, and policymakers but not on the perspectives of people living with dementia (Calabrese et al., 2026).

Guided by this conceptual framework, this study aims to explore how people living with dementia perceive and make sense of everyday interactions that can be understood through the theoretical lens of epistemic injustice. Specifically, it examines whether and how participants’ accounts describe experiences consistent with manifestations of epistemic injustice in their interactions with family members, healthcare professionals, and others involved in their everyday lives.

## Method

### Study design and participants

Semi-structured qualitative interviews were conducted with people living with mild dementia, either at home or in nursing homes, in Emilia-Romagna Region (Italy) between February 2025 and August 2025. Participants were aged 65 years or older, had been diagnosed with mild dementia, and were aware of their diagnosis. The inclusion criteria identified for communicating with professionals who collaborated in recruiting participants were a score on the Clinical Dementia Rating Scale (Hughes et al., 1982) equal to or more than one, but this was not an exclusion criterion: participants with CDR scores more than one but were assessed as being able to sustain the interview also were included. The organizations and associations involved were informed in advance of the inclusion criteria so that they could identify people who met criteria. The number of participants was determined based on the principle of theoretical saturation (Saunders et al., 2018): interviews were collected until new themes emerged. Once the themes were repeated, the interviews were discontinued. The research thus involved the collection of 15 interviews. Participants were recruited on a voluntary basis through a network of Italian associations and organizations involving people with mild dementia, as well as through referrals. Sampling followed a convenience approach based on willingness to participate. The study was approved by the Bioethics Committee of University of Bologna in December 2024 (Reference number: 0391787). Written informed consent was obtained from all participants.

### Interview guide

The interview questions were developed collaboratively by all authors, drawing on their professional experience and on the conceptual literature on epistemic injustice in healthcare and mental health settings (Carel & Kidd, 2014; Crichton et al., 2017; Kidd et al., 2022). The interview guide was shared in advance with professionals working in the organizations and associations involved in participant recruitment. It was specified that the guide was not intended to be followed rigidly and that a conversational and flexible approach to the interviews was preferred.

Participants were not introduced to the concept or terminology of epistemic injustice prior to the interviews. Instead, the interview guide explored concrete everyday experiences corresponding to its main manifestations—including whether participants felt listened to, whether their views were taken seriously, and whether they were included in conversations and decisions. These domains were selected because they reflect key dimensions of epistemic injustice described in the literature, namely recognition as a credible knower, opportunities for epistemic participation and involvement in shared processes of communication and decision-making (Carel & Kidd, 2014; Fricker, 2007; Kidd & Carel, 2017a). This approach allowed participants to describe their experiences in their own words without imposing a predefined conceptual framework. The aim was therefore not to ask participants whether they had experienced epistemic injustice, but to explore everyday interactions through which epistemic injustice might be enacted and subsequently interpreted within Fricker’s theoretical framework.

Examples of interview questions included:

1. Would you like to tell me how you felt after receiving your dementia diagnosis? Who gave you the diagnosis? Do you think it was communicated clearly and appropriately? Did you have the opportunity to ask questions about your diagnosis?
2. Do you feel that you have been treated differently by healthcare professionals or caregivers since receiving your diagnosis? If so, in what way? Can you think of any examples?

### Data collection

Before the interview began, written informed consent to participate in the study was obtained, and socio-demographic data were collected, including age, gender, level of education, and marital status.

Interviews were initially estimated to last approximately 1 hr; however, their duration varied considerably depending on the participant. While few interviews reached 1 hr; most lasted around 30 min. All interviews were conducted in person by researcher L.C., either at participants’ homes or nursing homes, or at the premises of the collaborating associations and organizations. Interviews took place in a quiet setting, without the presence of caregivers or healthcare professionals.

Conducting the study also presented several methodological challenges that are common in qualitative research involving people living with dementia. First, recruitment required substantial time and effort, as identifying healthcare professionals and community organizations willing to collaborate and support participant recruitment proved challenging. Furthermore, recruiting individuals with early-stage dementia was particularly difficult. In Italy, as in many other countries, dementia is frequently diagnosed at relatively advanced stages, when cognitive and communication impairments may substantially limit opportunities to participate in qualitative interviews (Alzheimer’s Disease International, 2024). Consequently, considerable effort was invested in identifying participants who were able to provide informed consent and meaningfully engage in an in-depth interview.

All interviews were audio-recorded with participants’ consent and subsequently transcribed verbatim by researcher L.C.

### Data analysis

Interview transcripts were analyzed using inductive thematic analysis following Braun and Clarke’s (2006) approach. Relevant phrases were identified, coded, and organized into units of meaning, which were subsequently grouped into broader categories of meaningful concepts. Audio recordings and transcripts were uploaded to a secure shared folder accessible to three authors (L.C., M.B., and R.C.).

Two researchers (L.C. and M.B.) performed the initial coding of all transcripts and subsequently independently identified the themes. In cases of disagreement, a third author (R.C.) was consulted until consensus was reached. The resulting themes were compared and discussed with the research team.

Throughout the analytic process, reflexivity was maintained by encouraging researchers to critically reflect on their own assumptions, disciplinary backgrounds, and potential influence on data interpretation (Braun & Clarke, 2006; Finlay, 2002). Reflexive discussions were integrated into team meetings to enhance transparency and analytical rigor (Lincoln & Guba, 1985). Although participants described concrete everyday experiences rather than using the language of epistemic injustice, data interpretation was theoretically informed by Fricker’s (2007) conceptual framework. Particular attention was paid to narratives describing diminished credibility, exclusion from conversations or decision-making, dismissal of participants’ contributions, or experiences in which participants were treated as passive recipients rather than knowledgeable agents. Accordingly, themes were interpreted as reflecting epistemic injustice only when they concerned participants’ exclusion from knowledge exchange or the denial of their epistemic agency, rather than social exclusion more broadly.

### Ethical considerations

During transcription, each participant was assigned a unique identification code at the time of enrolment, and all personal references were replaced with pseudonyms to ensure anonymity. Data were analyzed and reported in aggregate form.

## Results

Fifteen semi-structured interviews were conducted: six with people living in nursing homes and nine with people living at home. The variables were not compared because this was not an objective of our study. Other socio-demographic information about participants, such as age, gender, level of education, and marital status is included in Table 1.

**Table 1:** Socio-demographic characteristics of participants.

Variable	Mean or *n* (%)
**Age (years)**	80.1
**Sex**	
Female	10 (66.7)
Male	5 (33.3)
**Level of education**	
5 years	8 (53.3)
8 years	2 (13.3)
≥13 years	5 (33.3)
**Marital status**	
Married/civil partnership	8 (53.3)
Divorced	2 (13.3)
Single	3 (20.0)
Widowed	2 (13.3)
**Living situation**	
Lives alone	1 (6.7)
Lives only with partner	7 (46.7)
Lives with family	1 (6.7)
Lives in a nursing home	6 (40.0)

The aim of this study is to explore whether and how people living with dementia describe everyday experiences that can be understood through the theoretical lens of epistemic injustice, and how these experiences may relate to different aspects of their daily lives.

Three domains of epistemic injustice were identified: (a) Epistemic marginalization in clinical encounters, (b) Everyday negotiation of competence and agency, and (c) Epistemic invalidation and silencing in interpersonal relationships. These are the three main themes identified in our analysis. Each theme includes subthemes which will be included in Table 2.

**Table 2:** Summary of themes and subthemes that emerged from the thematic analysis.

Themes	Subthemes
**Epistemic marginalization in clinical encounters**	Communication
	Perception of limited interest (lack of validation)
	Reduced opportunities to contribute to decisions and express one’s own perspective
	Three-way conversation including a caregiver
	Desire to ask questions or express doubts but difficulty in doing so
**Everyday negotiation of competence and agency**	Perceived lack of autonomy in everyday life
	Sense of being replaced by caregivers and avoidance of tasks that could still be performed independently
	Positive feelings associated with acting independently
**Epistemic invalidation and silencing in interpersonal relationships**	Reduced sense of importance in social and family roles
	Reduced opportunities for epistemic participation through social isolation
	Choice not to disclose the diagnosis to friends/relatives
	Reduced conversational participation and redirection of communication toward caregivers
	Perceived lack of interest from others during conversations

### Epistemic marginalization in clinical encounters

Accounts within this theme described relationships with healthcare professionals as complex and ambivalent, characterized by both reassurance and discomfort. Communication with doctors was often perceived as technically competent but emotionally challenging. The following quotation illustrates such an experience:

The doctor is very competent, but he worries me a little. I discovered that I’m not well, that I have problems, and by doing so, he highlighted them even more (…), but he also comforted me. (Man, 79 years, living at home)

Within these accounts, the diagnostic process was described as both clarifying and unsettling. Some participants also suggested that receiving a dementia diagnosis coincided with perceived changes in the way healthcare professionals interacted with them. One participant described this perceived change as follows:

[The relationship is] not like before, because even the doctor doesn’t treat me like someone who is well. (Man, 67 years, nursing home)

Another recurrent aspect of this theme concerned a perceived lack of interest from healthcare professionals during clinical encounters. Appointments were often described as brief and focused on administrative tasks rather than interpersonal engagement. The following quotations illustrate these experiences:

They write a few notes and send you away. (Man, 73 years, living at home)

They don’t even look at me because they know I’ll end up there anyway. They don’t really care that much. (Man, 84 years, nursing home)

Accounts within this theme also described a predominantly passive role during clinical encounters and, in some cases, in everyday life. Rather than portraying active participation in decision-making, these narratives often reflected reliance on medical recommendations without feeling directly involved in the decision-making process. One participant explained:

I follow what the doctors told me to do. (Woman, 94 years, nursing home)

Similarly, one account described daily routines as flexible but largely unstructured, with limited encouragement to engage in meaningful activities:

They don’t make us do anything. If you want to stay in bed, they let you stay in bed; if not, you get up. If they need you, they call you, in my opinion, but if not, they don’t make you work. (Woman, 87 years, nursing home)

Accounts of clinical encounters also highlighted the frequent involvement of caregivers, particularly spouses, in conversations with healthcare professionals. In some narratives, participants themselves described encouraging clinicians to speak with their caregiver because of concerns about memory. One participant explained:

I tell them to speak to my wife. I may not remember, or I may remember wrong, so I tell them to speak to her. (Man, 79 years, living at home)

By contrast, other accounts described situations in which clinicians increasingly directed communication toward caregivers rather than participants themselves. One participant stated:

They speak more and more to my wife. (Man, 79 years, living at home)

The analysis also identified accounts describing a desire to ask questions or express doubts, alongside difficulties in doing so. Some narratives suggested that participants felt intimidated by doctors and preferred to seek clarification from family members. One participant explained:

I feel more comfortable asking my family for help because I feel more intimidated by doctors. (Woman, 94 years, nursing home)

Other accounts reflected attempts to ask questions despite experiencing difficulty in expressing them effectively. For example:

Yes, I feel free to ask questions, even though sometimes I try but fail. (Woman, 74 years, nursing home)

### Everyday negotiation of competence and agency

Accounts within this theme concerned participants’ experiences of autonomy, assistance, and their perceived ability to continue performing everyday activities. Although reduced autonomy or dependence does not in itself constitute an epistemic issue, some accounts became relevant to the present analytical framework when they concerned participants’ own assessments of what they remained able to do.

Participants described restrictions in everyday life, including limitations in freedom of movement and daily choices. One participant living in a nursing home described this experience as follows:

Living here is living in a constrained way, and I can’t even explain how I feel, but I feel… I feel bound. (Man, 67 years, nursing home)

Taken on its own, this account primarily describes a sense of constraint rather than an explicitly epistemic experience. Within the broader theme, however, experiences of limited autonomy provided an important context for understanding participants’ opportunities to exercise and communicate their own preferences and assessments in everyday life.

A more specific aspect concerned situations in which participants distinguished between the support they received from caregivers and their own perceptions of what they remained capable of doing. One participant described this as follows:

The things she does… My wife does them better than me, even though I could do them too, but she’s better at it. She remembers how to do lots of things, so she does them, but I could do them too. (Man, 79 years, living at home)

This account illustrates a potential discrepancy between the assistance provided within everyday care arrangements and the participant’s own assessment of his remaining abilities. While the participant acknowledged his wife’s greater competence in some activities, he also explicitly maintained that he remained capable of performing them himself. Within the interpretative framework adopted in this study, the epistemic significance of this account lies not in the provision of assistance itself, but in the potential reduction of opportunities for participants’ own judgements about their capabilities to be exercised and recognized.

Other accounts similarly highlighted the significance participants attached to acting independently and successfully managing everyday situations without assistance. One participant recalled:

I was about to call my wife to ask her to help me, but she didn’t hear me. So, I tried again and managed it on my own. Thank goodness, otherwise I would have made a fool of myself (…) I was glad she didn’t hear me this time. (Man, 82 years, living at home)

In this account, independently completing the task appeared to be associated not only with practical autonomy but also with the possibility of testing and confirming one’s own abilities. Rather than suggesting that independence is inherently epistemic, these accounts highlight the potential importance of everyday opportunities to exercise personal judgment and competence. Within this theme, the relationship between assistance and agency therefore emerged as complex: support could be necessary and valued while opportunities to act independently remained meaningful for participants’ perceptions of their own capabilities.

### Epistemic invalidation and silencing in interpersonal relationships

Accounts within this theme described a reduced sense of importance in social and family roles. These experiences became epistemically relevant when narratives referred to being progressively less involved in conversations, consulted less frequently, or perceived as less able to contribute meaningful perspectives within interpersonal relationships. Changes in family communication emerged as one context in which these experiences were described, particularly in relation to adult children. One participant illustrated this perceived change as follows:

In my opinion, they treat me a little differently (…). Now that my sons have realised that I have problems, they talk almost exclusively to my wife. (Man, 82 years, living at home)

Another recurrent aspect of this theme concerned changes in social relationships, including the loss or absence of friendships. Although the loss of social relationships is not, in itself, an instance of epistemic injustice, several accounts described these changes as reducing opportunities to participate in conversations, exchange experiences, and contribute to shared understanding, thereby limiting opportunities for epistemic participation. The following quotations illustrate these experiences:

Almost all of them have disappeared. (Man, 86 years, living at home)

Another participant reflected:

There was some collaboration. Now, instead, I’m here. (Man, 79 years, living at home)

Accounts also described broader changes in interpersonal relationships. As one participant explained:

Here, it is precisely the relationship with others that has changed somewhat. That is what annoys me. (Woman, 66 years, living at home)

In a large number of accounts, participants described having no social network. One participant stated:

I have no friends or relatives. (Woman, 74 years, living at home)

Within the interpretative framework adopted in this study, these accounts suggest that social isolation could reduce the relational contexts in which participants were able to share experiences, have their perspectives acknowledged, and maintain their role as contributors to shared knowledge.

Another aspect of this theme concerned decisions not to disclose the diagnosis to friends or relatives. Rather than being presented solely as a matter of privacy, some accounts described this choice as a response to anticipated disinterest or discomfort from others. One participant explained:

I don’t say I have memory problems because others don’t want to know (…). They don’t want to know because they don’t have them. (Woman, 94 years, nursing home)

Within these narratives, reluctance to disclose the diagnosis appeared to reflect expectations that personal experiences would not be understood, taken seriously, or regarded as meaningful by others.

Accounts within family relationships also described a lack of direct communication and difficulties sustaining conversations. In several narratives, family members were perceived as increasingly directing communication toward caregivers rather than participants themselves. One participant stated:

In my opinion, they treat me a little differently (…). Now that my sons have realised that I have problems, they almost only talk to my wife. (Man, 79 years, living at home)

Some accounts also described difficulties sustaining conversations with family members. Although these difficulties may partly reflect cognitive changes associated with dementia, the narratives frequently situated them within broader relational changes in which family members increasingly redirected conversations toward caregivers or appeared to assume that participants had little meaningful contribution to make. Within this interpersonal context, conversational difficulties were experienced as reinforcing feelings of epistemic exclusion rather than reflecting cognitive impairment alone. One participant reflected:

I have no topics of conversation; I have nothing to talk about with my sons. (Man, 86 years, living at home)

Accordingly, this quotation was interpreted not as evidence of epistemic injustice in isolation, but as part of a broader pattern in which participants described diminished opportunities to participate as valued conversational partners within family relationships.

Other accounts described experiences of being implicitly excluded from dialogue. The following quotations illustrate these experiences:

In a way, they don’t talk to me because they take it for granted that I’m not the same person I used to be. (Man, 84 years, living at home)

They rely on what my wife says. I can’t hold a conversation; I don’t know what to say anymore… (Man, 79 years, living at home)

Taken together, these accounts were interpreted as describing a progressive shift in epistemic credibility, whereby caregivers increasingly became the preferred source of information, even when participants wished to contribute their own perspectives.

Finally, accounts within this theme also described a perceived lack of interest from others during conversations, often expressed through interruptions or conversational disregard. One participant described this experience as follows:

Some people are patient and some are not, and some just interrupt and carry on with their own conversations without even caring about what I wanted to say. (Woman, 66 years, living at home)

Repeated experiences of being interrupted or overlooked during conversations were interpreted as limiting opportunities to communicate personal experiences and have one’s contributions recognized by others, illustrating everyday forms of epistemic exclusion.

Table 2 provides a summary of the themes and subthemes that emerged.

## Discussion

This study explored how people living with dementia described everyday experiences that can be understood through the theoretical lens of epistemic injustice, focusing on the domains in which these experiences emerged and their perceived implications for everyday life. Results indicate three main areas: epistemic marginalization in clinical encounters, everyday negotiation of competence and agency, and silencing in interpersonal relationships. Although analytically distinct, these three themes collectively illustrate how participants described progressively fewer opportunities to contribute as credible knowers across different contexts of everyday life.

Notably, participants expressed gratitude for others managing their affairs, framing this as necessary support. This pattern was particularly evident within the theme everyday negotiation of competence and agency, which highlighted the complex relationship between receiving assistance and maintaining opportunities to exercise and communicate one’s own judgment about remaining abilities. Participants could acknowledge and value the support provided by caregivers while also describing the importance of being able to act independently and of having opportunities to demonstrate what they believed they could still do. Such findings suggest that supportive practices may, in some circumstances, coexist with reduced opportunities for people living with dementia to exercise or have their own assessments of competence recognized. As Fricker (2007) argues, this process shapes individuals’ self-conceptions; in dementia, reduced opportunities to exercise and confirm one’s own competence may have implications for epistemic confidence and individuals’ perceptions of their own abilities (Bartlett, 2014; Kontos & Grigorovich, 2018b).

The findings relating to epistemic marginalization in clinical encounters further reinforce this interpretation. Participants described consultations in which communication was often redirected toward caregivers, opportunities to ask questions were experienced as difficult, and interactions with healthcare professionals were sometimes perceived as lacking genuine interest in their perspectives. These results align with literature positioning people living with dementia as unreliable knowers, particularly in clinical settings where their testimonies are discounted (Carel, 2017; Schwab et al., 2021). The findings also resonate with psychiatric research, where patients are often excluded from interpretative processes concerning their own experiences (Kidd et al., 2022). In dementia, cognitive impairment is frequently equated with a global loss of competence, leading to systematic exclusion (Carel & Kidd, 2014).

Importantly, the findings also highlight the need to distinguish cognitive impairment itself from epistemic injustice. Our findings do not suggest that every difficulty described by participants should be interpreted as an instance of epistemic injustice. Some experiences, such as difficulties sustaining conversations or recalling information, may reasonably reflect the cognitive consequences of dementia. Similarly, reduced autonomy, dependence on others, or the need for assistance do not in themselves constitute epistemic injustice. Rather, our analysis indicates that epistemic injustice emerges when these cognitive changes are accompanied by relational responses that systematically diminish opportunities for people living with dementia to contribute their own perspectives, exercise judgment, or be recognized as credible knowers. In the context of the second theme, this distinction is particularly important: the epistemic dimension does not arise from receiving assistance itself, but may arise when opportunities to exercise or have recognized one’s own judgment regarding personal abilities are reduced. In this sense, the injustice lies not in the impairment itself but in the social interpretation and interpersonal consequences that follow from it.

The theme of epistemic invalidation and silencing in interpersonal relationships further illustrates this distinction. Participants described changes in family and social relationships that appeared to reflect not only cognitive changes associated with dementia but also the ways in which others responded to those changes, including impatience, conversational disengagement, redirecting communication toward caregivers, or overlooking participants’ contributions (Sabat & Harré, 1992). These experiences reinforce feelings of diminished epistemic value and undermine wellbeing by impacting autonomy (Hydén, 2014).

Across the three domains identified, a common thread was the progressive erosion of participants’ epistemic agency. Whether in clinical encounters, everyday interactions involving assistance and personal competence, or family relationships, participants described experiences in which opportunities to express preferences, exercise personal judgment, demonstrate abilities, or contribute to conversations could become limited. These findings suggest that epistemic injustice should be understood not only as a matter of credibility deficits, but also as a gradual reduction in opportunities to participate in shared processes of meaning-making and decision-making.

Although the three themes concern different contexts, they appear to describe a common trajectory. In clinical encounters, participants described being progressively less involved in conversations and healthcare decisions; in everyday life, some accounts highlighted the importance of retaining opportunities to exercise personal judgment and demonstrate remaining abilities alongside the support provided by others; and within family and social relationships, they reported diminished opportunities to contribute to conversations and shared understanding. Considered together, these findings suggest that epistemic injustice is not expressed through isolated events but through the cumulative reduction of opportunities to participate as credible knowers across multiple domains of everyday life.

Crucially, participants rarely described these experiences as intentionally harmful. Instead, many accounts suggested that these interactions were often perceived as well-intentioned or motivated by protection and support. This was particularly apparent in the context of everyday assistance, where support could be both necessary and valued while opportunities for independent action and the exercise of personal judgment remained meaningful. This suggests a need for reflection on a socio-institutional system that normalizes exclusion. As Toebes (2023) notes, legal frameworks and risk-averse care models often institutionalize substitute decision-making. This systemic pattern shapes the self-understanding of people living with dementia, highlighting the urgency of care practices that preserve epistemic agency (Kontos & Grigorovich, 2018b; Zeilig et al., 2014).

These dynamics overlap with the concept of stigma (Corrigan et al., 2005). While stigma describes broader social devaluation (Sabat, 2001), epistemic injustice focuses on how this discrediting is enacted in concrete interactions through the undermining of credibility (Carel & Kidd, 2014; Fricker, 2007). Both lenses offer complementary perspectives on the lived experience of dementia.

Taken together, the three themes identified in this study suggest that epistemic injustice is not confined to isolated clinical interactions but permeates multiple domains of everyday life. Participants’ accounts illustrate how apparently ordinary practices—such as redirecting conversations to caregivers, making decisions on behalf of the person, or overlooking their contributions—may cumulatively undermine their epistemic agency. In the domain of everyday assistance, the findings further suggest that the relationship between support and epistemic agency is not straightforward: assistance may be necessary and positively valued while opportunities to exercise one’s own judgment and demonstrate remaining abilities may still require careful attention. Recognizing these subtle and often unintentional forms of exclusion is an essential step toward developing dementia care that genuinely supports participation, personhood, and inclusion.

### Strengths and weaknesses

The study has limitations. The small, context-specific sample limits transferability. Experiences may vary by dementia stage, socio-cultural background, and living situation—specifically, the distinction between community-based and residential care was not analytically differentiated. Additionally, as a qualitative study, findings may have been influenced by the researcher-participant interaction. Moreover, the analysis and interpretation of qualitative data are inherently shaped by the researchers’ perspectives, assumptions, and reflexive engagement with the data. Although reflexive practices were adopted throughout the study, complete neutrality cannot be achieved, and alternative interpretations of participants’ accounts remain possible (Braun & Clarke, 2019; Lincoln & Guba, 1985). Finally, the study focused exclusively on people living with dementia. While this centers marginalized voices, future research should include caregivers and professionals to examine how injustice is negotiated within relational contexts.

Despite these limits, a primary strength is the active involvement of people living with dementia as primary participants, rather than relying on proxy accounts. This aligns with rights-based research approaches. Furthermore, the semi-structured, conversational nature of the interviews allowed participants to articulate complex experiences in their own terms. In a context of frequent exclusion, creating these research spaces constitutes a deliberate counter-practice to epistemic injustice.

### Future perspectives

Future research should compare home-based and institutional settings to better understand how different care contexts shape opportunities for epistemic participation and to inform context-sensitive interventions. Further studies should also explore the perspectives of family caregivers and healthcare professionals to examine how they perceive communication, decision-making, and the involvement of people living with dementia, thereby providing a more comprehensive understanding of the interpersonal dynamics underlying epistemic injustice. Combining interviews with observational methods could additionally capture how epistemic agency is enacted in everyday interactions, including among people living with more advanced dementia. At an applied level, future research should develop and evaluate educational and training programs for healthcare professionals and family caregivers that promote epistemic recognition, effective communication, and shared decision-making. Such initiatives could support a shift from substitute decision-making toward care practices that actively preserve and foster the epistemic agency and participation of people living with dementia.

## Conclusions

This study suggests that many everyday experiences described by people living with dementia can be understood through the theoretical lens of epistemic injustice. Rather than arising from isolated acts of discrimination, participants’ accounts point to subtle and often well-intentioned interpersonal and institutional practices that may progressively limit opportunities to participate as credible knowers in clinical encounters, everyday decision-making, and family relationships.

Importantly, participants frequently described these experiences as understandable or even necessary, highlighting how epistemic exclusion may become normalized and internalized over time. This finding reinforces the importance of distinguishing the cognitive consequences of dementia from the social processes through which people may be denied opportunities to contribute their knowledge, preferences, and perspectives.

Together, these findings support the value of epistemic injustice as an interpretative framework for understanding the lived experience of dementia and highlight the need for care practices, research approaches, and policy frameworks that actively recognize and sustain the epistemic agency of people living with dementia.

## Data Availability

Due to the qualitative nature of this study and the need to protect the privacy and confidentiality of the participants, the interview transcripts and related materials are not publicly available or accessible to other researchers for replication purposes. Furthermore, the studies reported in this manuscript were not pre-registered.
